# Leveraging human-trained neural networks for cross-species chromatin regulation annotations

**DOI:** 10.1093/g3journal/jkag230

**Published:** 2026-09-02

**Authors:** Noémien Maillard, Julie Demars, Raphaël Mourad

**Affiliations:** GenPhySE, Université de Toulouse, INRAE, ENVT, Castanet-Tolosan F-31326, France; GenPhySE, Université de Toulouse, INRAE, ENVT, Castanet-Tolosan F-31326, France; MIAT, Université de Toulouse, INRAE, Castanet-Tolosan F-31326, France; University of Toulouse, UPS, Toulouse 31062, France

**Keywords:** chromatin accessibility, histone marks, transcription factors, livestock species

## Abstract

Analogous to the Encyclopedia of DNA Elements (ENCODE) project, the Functional Annotation of ANimal Genomes (FAANG) consortium has produced chromatin annotations for domesticated animals, albeit in smaller amounts. Although acquiring experimental data is more accessible and affordable for many species, human and mouse organisms will remain the reference. Classical methods based on sequence conservation can be used to infer missing annotations, but are inappropriate for nonconserved sequences. While regulatory sequences share low-to-moderate conservation, they have retained their regulatory function during the evolution process. Here, we take advantage of 3 neural networks (DeepBind, DeepSEA, and Enformer) trained with human and mouse ENCODE data to infer chromatin annotations (transcription factors binding, chromatin accessibility, and histone marks) in cattle, pig, chicken, and European seabass. For this purpose, we comprehensively assessed the quality of predictions using experimental data from FAANG, through area under the receiver operating characteristic curve and area under the precision–recall curve metrics. Our results showed low variability between tissues and similar performances for various annotations in mammals and chickens, with area under the precision–recall curve ranging from 0.663 ± 0.010 (chicken) to 0.765 ± 0.009 (pig) for the best predicted experiment, H3K4me3 (all tissues grouped), but lower (0.238 ± 0.005) in fish. Further analyses focused on pigs highlighted (i) accurate predictions even for nonconserved sequences and (ii) variable predictions depending on genomic feature annotations. Our results advocate the widespread use of human-trained neural networks as a first step in cross-species genome annotation before training species-specific models.

## Introduction

The chromatin, the complex between the DNA double helices and proteins, regulates the organization of DNA in 3 dimensions within the nucleus over time. Interactions between transcription factors (TF), insulators, histones, and their modifications, and DNA modulate chromatin condensation, leading to a continuum of DNA accessibility degrees that participate in gene expression regulation (Klemm et al. 2019). Therefore, the characterization of chromatin regulation is required to improve the annotation of genomes for a better understanding of noncoding genetic variation in complex traits (Ward and Kellis 2012).

For this purpose, the Encyclopedia of DNA Elements (ENCODE) consortium was created in the 2000s in order to generate data and help elucidate how this regulation is modulated in model organisms, including human (ENCODE Project Consortium 2004) and mouse (Yue et al. 2014). To date, almost 8,000 human and 1,400 murine ChIP-seq of both TF and histone marks, DNase-seq, and ATAC-seq experiments are referenced in the ENCODE data portal (Dunham et al. 2012; Luo et al. 2020) (Supplementary Data 1). Later, in the 2010s, the Functional Annotation of Animal Genomes (FAANG) initiative was organized to collect and analyze similar chromatin regulation data for species of agronomic interest, such as chickens, cattle, and pigs (Andersson et al. 2015). Initial efforts were made to standardize both protocols and computational analyses across different species, rather than conducting a large number of experiments. Although the FAANG data portal is still in expansion through additional international projects (Clark et al. 2020), much less data are available for livestock species (https://data.faang.org/). To date, 2,288 pig ChIP-seq and ATAC-seq experiments have been referenced, including biological and technical replicates for a limited number of experimental conditions (eg experiment type, tissue, developmental stage) (Supplementary Data 1).

As well as continuing to conduct costly and time-consuming wet lab experiments across species, computational methods based on sequence conservation and pairwise or multiple whole-genome alignment have been used over the last 2 decades to transfer annotations from well-annotated to poorly annotated genomes (Hinrichs et al. 2006; Zhao et al. 2014). However, multiple works have shown that noncoding sequences, including regulatory elements, often show weak conservation signals, thereby limiting the use of conservation-based tools (Nelson and Wardle 2013; Wong et al. 2020).

As an alternative to conservation-based methods, deep learning methods have been trained with ENCODE data to predict annotations of chromatin regulation, and a growing number of models are now available (Chen et al. 2024). For example, transposable elements (Yan et al. 2020) and regulatory activity predictions (Kelley et al. 2018; Ji et al. 2021) can be detected from sequence information. On the one hand, models are becoming increasingly sophisticated to improve performance through precision and recall metrics, while on the other hand, they are often used for a similar purpose in the study of model organisms (Eraslan et al. 2019). Although chromatin regulation is conserved across large evolutionary distances (Woo and Li 2012; Pérez-Rico et al. 2017; Wong et al. 2020; Phan et al. 2025), few studies have evaluated the use of such novel tools for cross-species functional annotation by focusing on enhancer predictions (Chen et al. 2018; Minnoye et al. 2020; Hong et al. 2021; MacPhillamy et al. 2022). Despite the affordable acquisition of omics experimental data in a broad range of species, human and mouse remain the reference, reinforcing the usefulness of these organisms for cross-species studies, including livestock.

Here, we comprehensively assessed the ability of 3 artificial neural networks (DeepBind (Alipanahi et al. 2015), DeepSEA (Zhou et al. 2018), and Enformer (Avsec et al. 2021)), trained using human and mouse chromatin regulation data (ENCODE project, https://www.encodeproject.org/). Those neural networks share features, such as the use of convolution to detect predictive motifs and the predicted experiments. They are also complementary and represent 3 levels of the evolution of neural networks with their different input sequences, multiple convolution layers for DeepSEA and Enformer, and the addition of transformer layers for Enformer (Avsec et al. 2021). For fair comparisons, we used data available from the FAANG consortium (https://data.faang.org/). Our analyses include not only mammals (Chen et al. 2018) (mouse, pig, and cattle) but also chicken and seabass. Moreover, we did not limit our analysis to annotated regulatory sequences (Chen et al. 2018; Minnoye et al. 2020) (promoters and enhancers) but assessed the neural cross-species prediction at the whole-genome scale. We first compared the cross-species abilities of the different neural networks to predict a wide range of experiments. We then evaluated the performance of neural networks trained on human and mouse data to predict annotations across the phylogenetic tree and their limitations. Finally, we investigated the influence of sequence conservation and genomic features on the performance of chromatin prediction annotations.

## Materials and methods

### DeepBind model

DeepBind (Alipanahi et al. 2015) is a convolutional neural network taking 200 bp sequences as input. The output is a pseudoprobability, ranging from 0 to 1, that a given protein binds to the sequence. The network has been used to predict the binding of CCCTC-binding factor (CTCF), RB binding protein 5 (RBBP5), Nanog homeobox (NANOG), MAX, MYC, POU5F1, SIN3A, SUZ12, and YY1 with the corresponding models, respectively, D00328.018, D00797.001, D00786.001, D00504.005, D00785.001, D00795.001, D00804.002, D00812.001, and D00710.007. DeepBind predictions have been computed on the Genobioinfo cluster of the Genotoul-Bioinfo platform, parallelized on 64 CPU cores (approximately 11 h for the predictions with all 9 models on the whole mouse genome).

### DeepSEA model

DeepSEA (Zhou et al. 2018) is a convolutional-based model taking a 2,000 bp (context and sequence) as input, and which outputs a pseudoprobability, ranging from 0 to 1, to observe a peak in a 200 bp centered sequence. The model has been downloaded from Kipoi (Avsec et al. 2019). DeepSEA was used to predict 2002 experiments (DNase, ChIP-seq of TFs, and histone marks) for each sequence. TFs (CTCF, RBBP5, NANOG, MAX, MYC, POU5F1, SIN3A, SUZ12, and YY1), DNase, and histone ChIP-seq experiments (H3K4me1, H3K4me3, H3K27ac, and H3K27me3) from tissues of interest (liver [HepG2], lung [A549], muscle [HSMM]) have been selected. DeepSEA predictions have been computed with an NVIDIA RTX4090 GPU (prediction times ranging from 50 min for the seabass genome to 2h30 for the pig and cattle genomes).

### Enformer model

Enformer (Avsec et al. 2021) is a deep learning model composed of convolutional layers followed by transformer layers. It takes a 196,608 bp length sequence as input and predicts the centered 114,688 sequence as 896 bins of 128 bp length. Enformer predicts 5,313 human genomic tracks (DNase-seq, ChIP-seq of TFs and histone marks, ATAC-seq, and CAGE) for each sequence. We extracted ChIP-seq experiments of CTCF, H3K4me1, H3K4me3, H3K27ac, H3K27me3, DNase-seq, and ATAC-seq related to adipose, liver, lung, muscle, and spleen tissues. Enformer predicts a normalized read count. Enformer predictions have been computed with an NVIDIA RTX4090 GPU (prediction times ranging from 30 min for the seabass genome to 2h50 for the human genome).

### Data preparation for gkmSVM model

The dataset has been prepared with hESC CTCF data from goldenPath used to train the original DeepSEA (Zhou et al. 2018) model (Supplementary Data 2). Negative sequences have been created with default parameters (genomeVersion = “hg19”) from the BSgenome.Hsapiens.UCSC.hg19.masked (version 1.3.993) with a corrected version of the genNullSeqs function from gkvSVM (Ghandi et al. 2016) R library. We generated negative sequences with a ratio of 1.5 negative sequences for 1 positive sequence. For limited resources reasons, a quarter of the dataset has been used for training and testing the gkmSVM model.

### Training of gkmSVM

A gkmSVM model has been trained using the suggested pipeline from gkmSVM (Ghandi et al. 2016) R library (version 0.83.0). The gkmsvm_kernel, gkmsvm_train, and gkmsvm_classify functions have been used with default parameters (L = 10, K = 6, maxnmm = 3). The dataset detailed in the previous paragraph has been split into 70% data for training and 30% data for testing.

### Data preparation for humanDeepBind and mouseDeepBind models

For the human-trained model, the dataset has been prepared with hESC CTCF, RBBP5, and NANOG data from UCSC goldenPath (http://hgdownload.cse.ucsc.edu/goldenPath/) used to train the original DeepSEA (Zhou et al. 2018) model (Supplementary Data 2). For the mouse-trained model, the dataset has been prepared with mESC CTCF, RBBP5, and NANOG data lifted over mm39 mouse genome (GSE80280) with the LiftOver (Hinrichs et al. 2006) tool from UCSC (Supplementary Data 3). For both datasets, the same procedure has been followed. The BSgenome.Hsapiens.UCSC.hg19 (version 1.4.3) and the BSgenome.Mmusculus.mm39 (version 1.0.0) genomes have been used for human and mouse, respectively. The genomes have been binned into 200 bp bins; chromosomes and contigs in CTCF, RBBP5, and NANOG observed data have been conserved to compute bin overlapping; and bins have been classified as positive when half of the bin was overlapped by a TF peak; otherwise, it has been classified as negative. For human data, this represents 77,949 positive bins and 15,323,421 negative bins for CTCF; 48,470 positive bins and 15,352,963 negative bins for RBBP5; and 6,572 positive bins and 15,394,843 negative bins for NANOG. For mouse data, this represents 11,801 positive bins and 13,437,515 negative bins for CTCF; 9,694 positive bins and 13,084,618 negative bins for RBBP5; and 39,064 positive bins and 13,510,252 negative bins for NANOG.

### Training of humanDeepBind and mouseDeepBind

Both DeepBind models have the same architecture. It is a convolutional neural network with the same architecture as the original DeepBind model. It is composed of a convolutional layer with 16 filters of size 24 with ReLU activation function, followed by a global max pooling layer, a dense layer with 32 neurons, and ReLU activation with 0.5 dropout. The output is a dense layer with 1 neuron and linear activation, followed by a sigmoid activation to obtain pseudoprobabilities. The network has been trained with the Adam optimizer to minimize the binary cross-entropy loss function. The models have been trained on 1 NVIDIA RTX4090 GPU with TensorFlow 2.17.0 as the backend of Keras 3.6.0, for 29 epochs for the human model and 15 epochs for the mouse model, and a learning rate of 1.10^−3^ with a batch size of 100 sequences. The sequences have been one-hot encoded for A, C, G, T, and N. The script has been adapted from Raphaël Mourad's script on Google Colab (https://colab.research.google.com/drive/1WEfVpgzzpWwQ3hBGi4bV6NbuzCP_8dwV#scrollTo=OxW_pnHVv3Aa). The dataset detailed in the previous paragraph has been split into 70% data for training and 30% data for testing. Twenty percent of the training set has been used for validation.

### Mouse genome

Mouse reference genome (GRCm39, mm39) from Ensembl release 112 has been used to predict peaks with DeepBind (Alipanahi et al. 2015) and DeepSEA (Zhou et al. 2018). For this purpose, the genome has been binned into 200 bp nonoverlapping bins for both DeepBind (13,641,080 bins corresponding to 2,728,216,000 nucleotides) and DeepSEA (13,640,500 bins corresponding to 2,728,100,000 nucleotides), and then DNA sequences have been extracted according to bins. For DeepSEA, the bins have been resized to 2,000 bp for sequence extraction. For DeepBind, the truncated bins at the end of chromosomes were removed. For DeepSEA, the truncated bins at both ends of chromosomes were removed. Except for the nonpredicted chromosome ends, the binned genomes have no gap. For each sequence, the models were used to predict the pseudoprobability, ranging from 0 to 1, of binding for the different experiments.

### Mouse ChIP-seq and DNase-seq data

All mouse experimental data have been obtained in the embryonic stem cell line. ChIP-seq bed files of TFs (CTCF [GSE60917, GSE29218, GSE114599, GSE112132, GSE123101, GSE80280, GSE34094, and GSE25777], RBBP5 [GSE22934], NANOG [GSE44286, GSE59378, and GSE55404], MAX [GSE48175], MYC [GSE90893 and GSE43229], POU5F1 [GSE44286, GSE113913, GSE59378, and GSE102517], SIN3A [GSE81081 and GSE117287], SUZ12 [GSE127804, GSE103685, GSE60397, GSE72164, and GSE83094], and YY1 [GSE68195, GSE99518, and GSE140546]) have been extracted from ReMap2022 (Hammal et al. 2022) and bed files of DNase-seq and histone mark experiments have been extracted from ENCODE (DNase-seq [ENCFF178MGP], ChIP-seq H3K4me1 [ENCFF426IIV], ChIP-seq H3K4me3 [ENCFF247GVM], ChIP-seq H3K27ac [ENCFF360VIS], and ChIP-seq H3K27me3 [ENCFF008XKX]) (Supplementary Data 2). Biological replicates have been chosen with many peaks in the same order of magnitude than training data of DeepSEA (Zhou et al. 2018). The peaks have been lifted over the more recent genome mm39 with the LiftOver (Hinrichs et al. 2006) tool from UCSC (Supplementary Data 3).

### Area under the receiver operating characteristic curve and area under the precision–recall curve value extraction from publications

For human (GRCh37, hg19 for both DeepBind (Alipanahi et al. 2015) and DeepSEA (Zhou et al. 2018), respectively) ChIP-seq peak predictions, we extracted available area under the receiver operating characteristic curve (AUC-ROC) and area under the precision–recall curve (AUC-PR) values from publications. For DeepBind, all the AUC-ROC have been extracted from Supplementary Table 5 (41587_2015_BFnbt3300_MOESM56_ESM.xlsx) for each corresponding TF in the DeepBind* column. For DeepSEA, we downloaded the sei_vs_beluga_comparison.tar.gz archive in the download_data.sh file from the sei-manuscript GitHub repository (https://github.com/FunctionLab/sei-manuscript). Then we extracted AUC-ROC (roc_auc column) and AUC-PR (average_precision column) from the test performance of Beluga. The exact path from the downloaded file is available in Supplementary Data 3.

### AUC-ROC and AUC-PR computation

For class labeling, a bin has been considered positive (class 1) if at least half of the bin overlaps with an observed peak; otherwise, it has been considered negative. Multiple peak overlap with 1 bin led to 1 positive bin. For this, the findOverlaps function from the GenomicRanges (Lawrence et al. 2013) library was used (version 1.56.2) with default parameters (maxgap=-1L, ignore.strand = TRUE) except minoverlap set at half of the bin width (100L for DeepBind and DeepSEA and 64L for Enformer). AUC-ROC and AUC-PR have been computed using the roc.curve and pr.curve functions, respectively, from the PRROC (Grau et al. 2015) R library (version 1.3.1). For both functions, the prediction scores have been supplied for the corresponding classes. Note that, contrary to DeepBind and DeepSEA, which predict a pseudoprobability ranging from 0 to 1, Enformer predicts a normalized read count. However, the functions are agnostic to the type of scores since they only use them to order classed bins (encoded as zeros and ones). An AUC-PR baseline (ie prevalence) has also been computed for all experiments as the proportion of positive bins among all bins. For livestock species analysis, a random classifier has also been assessed. This classifier has been designed using the random number generator runif R-base (version 4.5.3) function, which generates random deviates between 0 and 1 (both excluded).

### Livestock species genomes

Pig reference genome (Sscrofa11.1), chicken reference genome (GRCg7b, bGalGal1), and European seabass reference genome (dlabrax2021) have been downloaded from Ensembl release 112. The cattle reference genome (ARS-UCD1.2, bosTau9) has been downloaded from Ensembl release 99. Each livestock species genome has been used to predict peaks with DeepSEA (Zhou et al. 2018) and Enformer (Avsec et al. 2021). Each genome has been binned into 200 and 128 bp nonoverlapping bins for both DeepSEA (12,503,419 bins corresponding to 2,500,683,800 nucleotides for pig genome; 13,557,236 bins corresponding to 2,711,447,200 nucleotides for cattle genome; and 5,264,516 bins corresponding to 1,052,903,200 nucleotides for chicken genome) and Enformer (19,123,328 bins corresponding to 2,447,785,984 nucleotides for pig genome; 20,563,200 bins corresponding to 2,632,089,600 nucleotides; and 8,031,744 bins corresponding to 1,028,063,232 nucleotides for chicken genome) predictions, respectively. Sequences have been extracted from bins resized to 2,000 bp bins for DeepSEA. For Enformer, sequences have been extracted from each subsequent block of 896 bins, resized to 196,608 bp. For both DeepSEA and Enformer, the truncated bins at both ends of chromosomes were removed. Except for the nonpredicted chromosome ends, the binned genomes have no gap, but due to its large receptive field, some scaffolds have not been predicted with Enformer. For each sequence, DeepSEA was used to predict the pseudoprobability to observe a peak, and Enformer predicted a normalized read count. For DeepSEA and Enformer predictions, AUC-ROC and AUC-PR have been calculated with the same method as for mouse experiments.

### Livestock species ChIP-seq and chromatin accessibility data

For each experiment, pig, chicken, and cattle data bed files have been downloaded from the FAANG consortium (Kern et al. 2021) (GSE158430). ChIP-seq (CTCF, H3K4me1, H3K4me3, H3K27ac, and H3K27me3), DNase-seq (chicken), and ATAC-seq (pig and cattle) performed in adipose, liver, lung, muscle, and spleen tissues have been chosen. European seabass bigBed of the same experiments in the lung and spleen have been downloaded from the AQUA-FAANG consortium (Dyer et al. 2025) (http://ensembl.org database), and then the bed files have been created from bigBed using bigBedToBed from UCSC (Supplementary Data 2). The bed files have been kept as is, except for the chicken data that were lifted over to the more recent genome bGalGal1 using the LiftOver (Hinrichs et al. 2006) tool from UCSC.

### Human ChIP-seq and DNase-seq data

Human CTCF (ENCFF478CPK), DNase (ENCFF252LJK), H3K4me1 (ENCFF983WBS), H3K4me3 (ENCFF036ODU), H3K27ac (ENCFF283JME), and H3K27me3 (ENCFF270WAT) in muscles have been downloaded from the ENCODE consortium. The data have been used for cross-species liftover using the LiftOver (Hinrichs et al. 2006) tool from UCSC and AUC-ROC and AUC-PR computation before computing pig AUC loss relative to human AUCs.

### Livestock species to human LiftOver

For CTCF, H3K4me1, and H3K4me3 in the muscle, pig, cattle, and chicken bed from the FAANG consortium (GSE158430) have been lifted over the hg38 human genome with the LiftOver (Hinrichs et al. 2006) tool from UCSC (Supplementary Data 3).

### Human to livestock species LiftOver

For CTCF (ENCFF478CPK), H3K4me1 (ENCFF983WBS), and H3K4me3 (ENCFF036ODU) in the muscle, human bed from ENCODE have been lifted over pig (Sscrofa11.1) and cattle (ARS-UCD1.2, bosTau9) genomes with the LiftOver (Hinrichs et al. 2006) tool from UCSC (Supplementary Data 3).

### Enformer peak calling

Bigwig predictions have been converted into bed peaks with a home-made algorithm to identify the threshold, classifying bins into positives and negatives, to obtain as many peaks as observed in humans. The number of expected peaks has been adjusted by the proportion of the predicted pig genome.

### Pig genome annotations

The GTF files of genomic feature annotations (gene, transcript, Coding Sequence [CDS], exon, 5′UTR, 3′UTR) and regulatory annotations (promoter, enhancer) have been extracted from Ensembl database release 115. Repeats have been extracted from the UCSC database. CpG islands have been extracted from the UCSC Table Browser. For conserved region analysis, the gerp_conservation_scores.sus_scrofa.Sscrofa11.1.bw file from the 91_mammals.gerp_conservation_score folder has been extracted from Ensembl release 113 (Dyer et al. 2025) (Supplementary Data 3).

### Human genome

To calculate AUC-ROC and AUC-PR differences from the human to the pig with Enformer (Avsec et al. 2021) predictions, the human reference genome (GRCh38, hg38) from Ensembl release 112 has been used. For this purpose, the genome has been binned into 128 bp nonoverlapping bins (24,734,976 bins corresponding to 3,166,076,928 nucleotides). Then, DNA sequences have been extracted from each subsequent block of 896 bins, resized to 196,608 bp. The truncated bins at both ends of chromosomes were removed. Except for the nonpredicted chromosome ends, the binned genome has no gap, but due to its large receptive field, some scaffolds have not been predicted with Enformer. For each sequence, the model was used to predict the normalized read count for the different experiments. AUC-ROC and AUC-PR have been calculated with the same method as for mouse experiments.

### Human genome annotations

The GTF files of genomic features (gene, transcript, CDS, exon, 5′UTR, 3′UTR) and regulatory annotations (promoter, enhancer) have been extracted from Ensembl database release 115. Repeats have been extracted from the UCSC database. CpG islands (CGIs) have been extracted from the UCSC Table Browser (Supplementary Data 3).

### NGS read alignments

For Integrative Genome Viewer (IGV) (Robinson et al. 2023) panels, mouse, pig, and chicken raw reads have been aligned on mm39, Sscrofa11.1, and bGalGal1 reference genomes, respectively. Mouse raw reads have been extracted with the SRA run selector for TFs and from the ENCODE consortium for H3K4me3 and DNase I hypersensitivity experiments. Pig and chicken raw reads have been extracted from data referenced in the FAANG consortium corresponding to GSE158430 (PRJEB14330, PRJNA665197, PRJNA665199, PRJNA665209, PRJNA665214, PRJNA665216, PRJNA665216). For ChIP-seq, inputs have been merged for each tissue-sample couple (Supplementary Data 4). For the alignment of raw reads, the nf-core ChIP-seq (for CTCF and H3K4me3 experiments) and ATAC-seq (for ATAC-seq and DNase-seq experiments) pipelines have been used with default parameters. The macs_fdr parameter has been set to 0.01 for ChIP-seq experiments and 0.05 for ATAC-seq/DNase-seq experiments.

### Integrative Genome Viewer

IGV (Robinson et al. 2023) has been used to show how predictions fit observations for genomic regions. For DeepSEA (Zhou et al. 2018), the pseudoprobabilities have been used. Predicted peak heights have been set with the autoscale parameter.

### Statistical information

Wilcoxon tests have been computed using a 2-tailed *wilcox_test* function from the coin (Hothorn et al. 2008) R library. The r effect size has been computed by dividing the Z statistic from the Wilcoxon tests by the square root of the sample sizes for each test.

### Artificial intelligence statement

The authors declare limited use of ChatGPT (GPT-4 Turbo to GPT-5.3) and Claude (Sonnet 4.6) for assisted code writing. Artificial intelligence-based writing assistance (Grammarly) was used solely to improve the grammatical accuracy and readability of the text.

## Results

### Experimental design of cross-species predictions

To investigate the relevance of human- and mouse-trained neural networks for translational studies, especially to identify genome-to-phenome links in livestock, we used 3 artificial neural networks presenting different properties (Supplementary Data 5) and trained on various biological experiments related to chromatin regulation (DeepBind (Alipanahi et al. 2015), DeepSEA (Zhou et al. 2018), and Enformer (Avsec et al. 2021)) (Fig. 1a; Supplementary Data 5). DeepBind and DeepSEA are based on convolution layers, while Enformer (Avsec et al. 2021) also uses transformer layers. In addition, the size of the input sequence ranges from 200 bp for DeepBind, 2 kb for DeepSEA, and up to 196 kb for Enformer (Fig. 1a; Supplementary Data 5). Cross-species predictions of functional chromatin annotations, including TF binding sites and histone marks obtained from ChIP-seq data, as well as chromatin accessibility obtained from ATAC-seq and DNase-seq data, were performed using the reference genomes of the mouse (mm39), pig (Sscrofa11.1), cattle (ARS-UCD1.2), chicken (bGalGal1), and European seabass (dlabrax2021) (Fig. 1b). The performance of the predictions was evaluated using standard metrics (AUC-ROC and AUC-PR) by comparing the predicted data with the observed data from ReMap2022 (Hammal et al. 2022) and the FAANG data portal (https://data.faang.org/) (Fig. 1c). AUC-ROC is classically used for benchmarks, but does not account for class imbalance (ie most bins do not overlap a peak). Hence, AUC-PR has also been used to account for class imbalance. Indeed, since peaks (the positive class) are rare, AUC-PR is more punishing of false positives in a sea of negatives than AUC-ROC is.

**Fig. 1.**
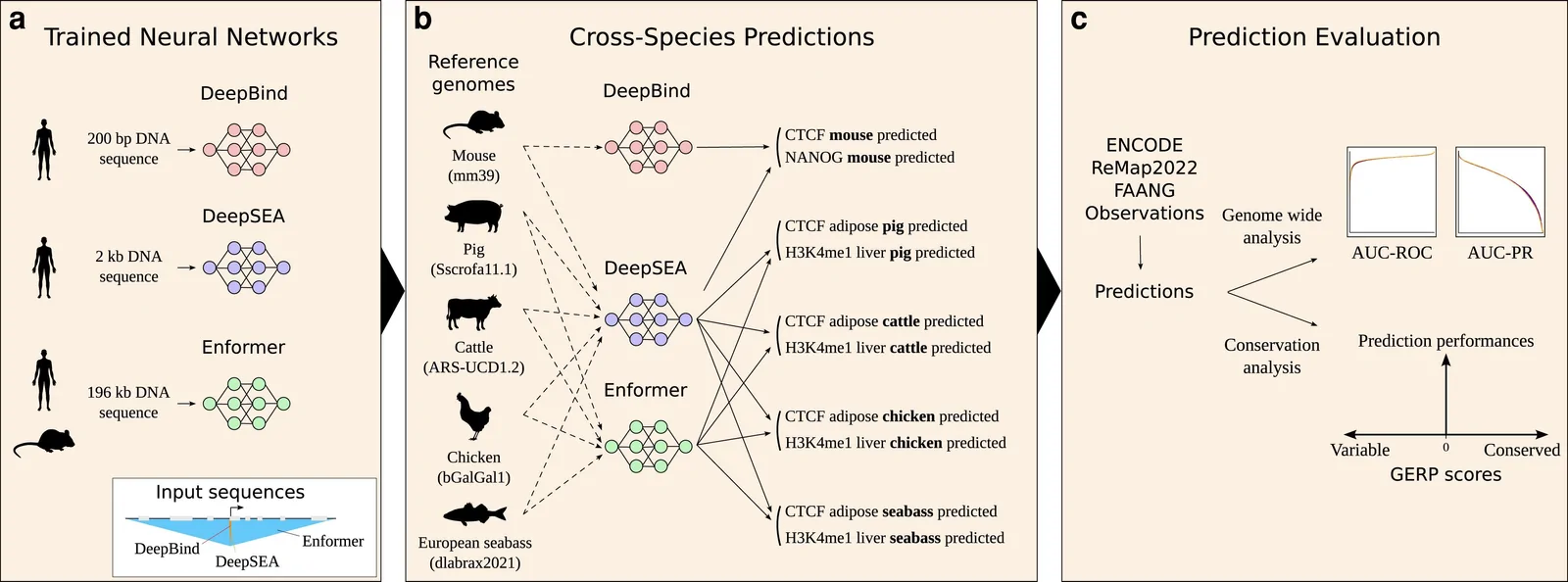
Approach to perform cross-species functional chromatin annotation predictions in livestock using human- and mouse-trained neural networks. a) Neural networks trained on human and mouse data. Three neural networks have been used: DeepBind (Alipanahi et al. 2015), DeepSEA (Zhou et al. 2018), and Enformer (Avsec et al. 2021). b) Cross-species predictions. Reference genomes of mouse (mm39), pig (Sscrofa11.1), cattle (ARS-UCD1.2), chicken (bGalGal1), and European seabass (dlabrax2021) have been used. c) Evaluation of prediction performances. Standard metrics (AUC-ROC and AUC-PR) were calculated by comparing the predicted data with the observed data from the ENCODE (https://www.encodeproject.org/), ReMap2022 (https://remap2022.univ-amu.fr/), and FAANG (https://data.faang.org/) databases. For a finer analysis, the GERP conservation scores calculated from 91 mammal genomes have been used to show whether only sequences with conserved nucleotides can be well predicted.

### Prediction of chromatin annotations in mouse using human-trained models

To evaluate the ability of neural networks trained with human data to predict diverse annotations on the genome of another mammal, we first predicted annotations from the mouse reference genome (mm39) using only DeepBind (Alipanahi et al. 2015) and DeepSEA (Zhou et al. 2018). Since Enformer (Avsec et al. 2021) has been trained using both human and mouse data, predicting experiments from the mouse genome would likely lead to data leakage. We also trained 3 models with human or mouse embryonic stem cells (ESC) CTCF data to use them for comparison: a gkmSVM model trained with human data and evaluated on human and mouse data, a human-trained DeepBind model evaluated on mouse and human data, and a mouse-trained DeepBind model evaluated on mouse data.

To validate the better performances of neural networks, we compared DeepBind (Alipanahi et al. 2015) and DeepSEA (Zhou et al. 2018) performances to the gkmSVM model performances. While the gkmSVM seems to predict CTCF binding well, the mouse AUC-ROC of 0.759 represents 18.8% loss of AUC-ROC compared to the human 0.935 AUC-ROC (Supplementary Data 6). In contrast, DeepBind loses only 6.3% AUC-ROC when predicting mouse data, and DeepSEA even gains 0.9% compared to evaluation on human data (Fig. 2a). When focusing on the AUC-PR, the gkmSVM shows 15.6% AUC loss, while DeepSEA cross-species predictions show 24.7% loss. However, as underlined by their respective AUC-PR baselines, the dataset used to train the gkmSVM model has a different prevalence (1 positive for 1.5 negative sequences) than the neural network models. This is because the gkmSVM cannot take advantage of GPU acceleration, so we had to adapt the training dataset, which could not be biologically meaningful. This shows a better scaling of neural networks and, as a consequence, better performances.

**Fig. 2.**
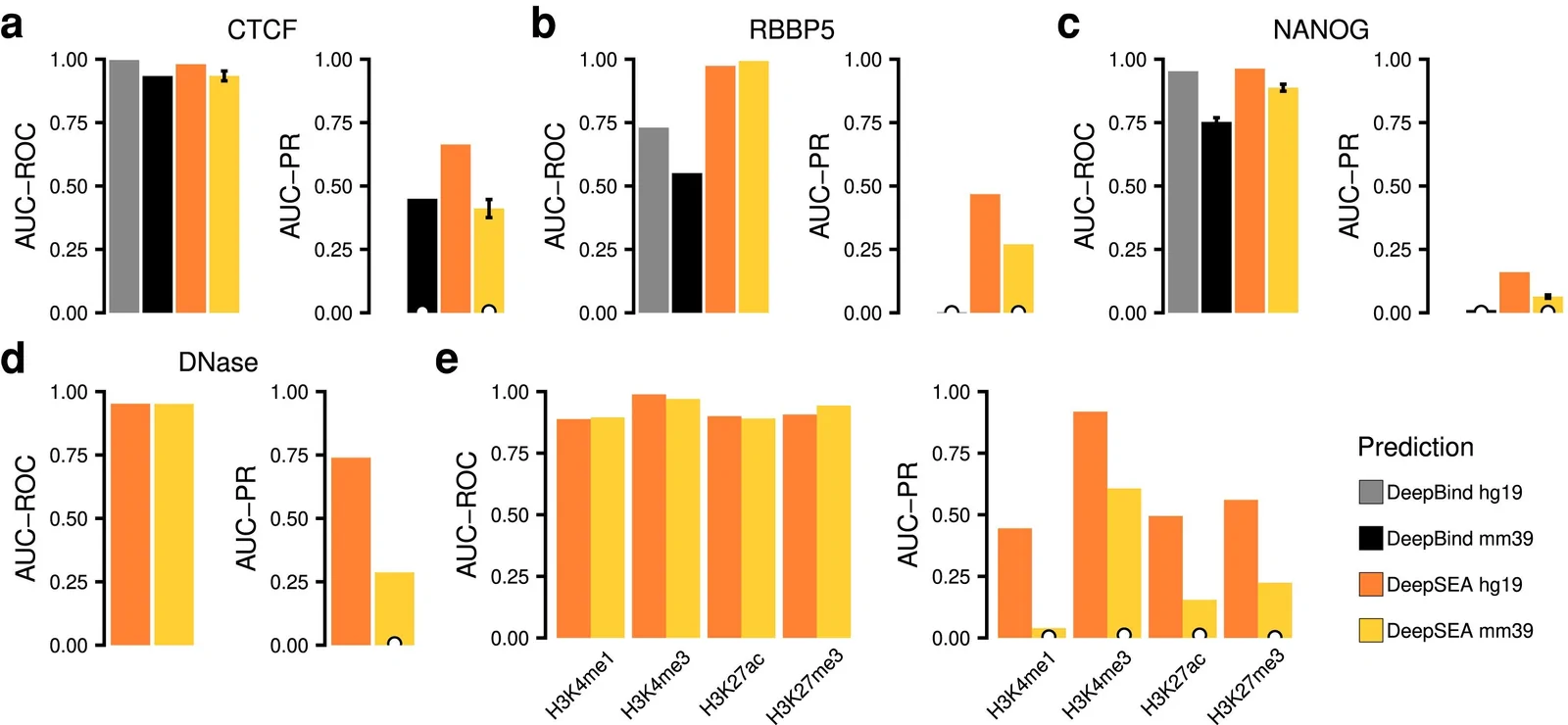
Predictive performances of 2 human-trained neural networks on the mouse genome. AUC-ROC (left) and AUC-PR (right) for CTCF a), RBBP5, histone lysine methyltransferase complex subunit b), NANOG c), DNase d), and histone marks e). The DeepBind prediction metric values are in gray and black for the human (hg19) and mouse (mm39) genomes, respectively. The DeepSEA prediction metric values are in orange and yellow for the human (hg19) and mouse (mm39) genomes, respectively. AUC-PR baselines are in white. The human AUC values have been extracted from the original articles for DeepBind (Alipanahi et al. 2015) and DeepSEA (Zhou et al. 2018). Except for the DeepBind-CTCF prediction (model trained with GM12801 cell line), which was compared to CTCF conducted in WEHI cell line, the observations used for comparisons with predictions were conducted on mouse embryonic stem cells (ESCs). For CTCF and NANOG, data shown (including AUC-PR baselines) are means ± SE (*n* = 8 and *n* = 3 for CTCF and NANOG, respectively; *n* = 1 for other experiments).

Because data are scarce for most of the species, and cross-species predictions show similar predictive ability to neural networks trained with data of the species of interest (Supplementary Data 7), cross-species predictions seem an attractive alternative to test for a broader range of experiments. As shown in Fig. 2a, predictions on the mouse genome showed high accuracy for CTCF peaks with AUC-ROC values of 0.934 and 0.940 ± 0.018 using DeepBind and DeepSEA, respectively, which were close to AUC-ROC values for the human genome (0.997 for DeepBind and 0.980 for DeepSEA). We observed that all AUC-PR, which accounts for class imbalance, showed higher values than their own reference AUC-PR baseline. For DeepSEA AUC-PR, we obtained a value of 0.412 ± 0.031 for mouse CTCF predictions, whereas it was 0.664 for human predictions (Fig. 2a). For all evaluated TFs, DeepSEA showed similar or better AUC-PR values than DeepBind (Fig. 2b and c; Supplementary Data 8). This also stands for AUC-PR/prevalence ratios (Supplementary Data 9). In addition, we also observed that ubiquitous TFs, including CTCF and RBBP5, and histone lysine methyltransferase complex subunit, showed higher AUC-PR values and AUC-PR/prevalence ratios than TFs in a different biological context, such as NANOG (Fig. 2a to c; Supplementary Data 8 and 9). As shown in Supplementary Data 10, observed data of human and mouse TFs show similar peak counts and peak widths, supporting genuine good cross-species performances, rather than dataset differences. For DNase and histone marks predicted only with DeepSEA, AUC-ROC values for mouse predictions were similar to those for human predictions, but there was a more significant gap between AUC-PR values between mouse and human predictions (Fig. 2d and e). While this can be partially a consequence of differences between human observed data used for training and mouse observed data used for evaluation (Supplementary Data 11), our results show better AUC-PR than the corresponding baselines (ie prevalence) (Fig. 2; Supplementary Data 8 and 9), demonstrating good cross-species performance.

Overall, the correlations between human and mouse AUC-PR values appeared to be stronger for TF binding sites, regardless of the AUC-PR values, than for histone and DNase experiments, despite the greater amount of peak sequence in the training data (Fig. 3a). Indeed, when training a machine learning model, it is expected that the amount of training data (here, the number of peak sequences) will strongly influence prediction performances (Liu 2022), because more training data would lead to a better representation of reality. In agreement, we observed a strong correlation between the AUC-PR or AUC-PR/prevalence ratio calculated with DeepSEA predictions on both human (Fig. 3b) and mouse (Fig. 3c; Supplementary Data 12) genomes and the number of peaks used to train the model. We then focused on the *Sox2* and *Igf2* loci, which are known to carry binding sites for NANOG and CTCF, respectively (Fig. 3d). For NANOG predictions, 1 true positive out of 4 was predicted in the *Sox2* region. However, a high error rate has also been measured with 3 false negatives and 1 false positive (Fig. 3d). Conversely, CTCF experimental peaks are well predicted in the *Igf2* region, albeit at a high false-positive rate (Fig. 3d). Like CTCF, DNase, and H3K4me3, observed peaks were correctly predicted despite false-positive peak predictions (Fig. 3d).

**Fig. 3.**
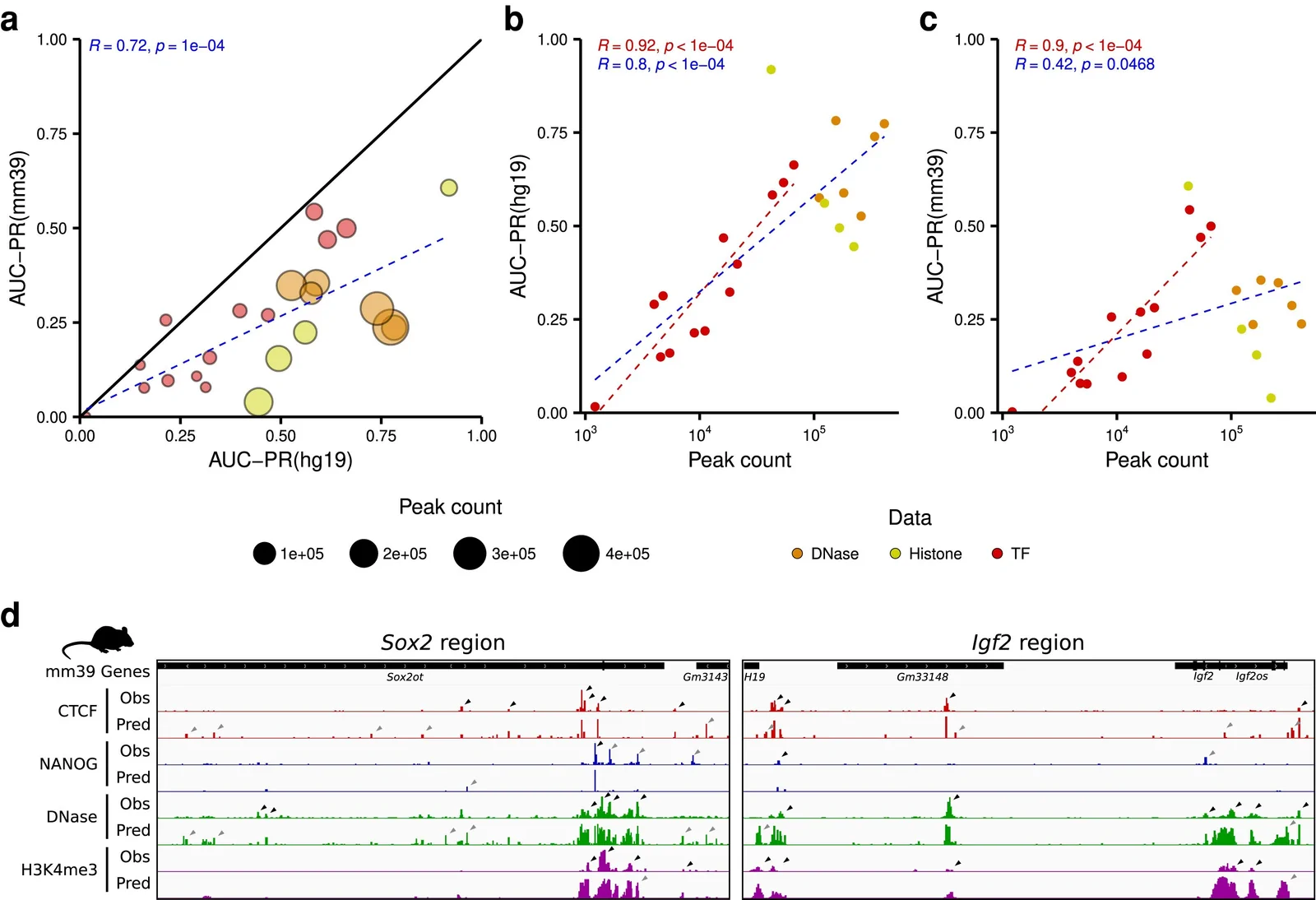
Comparison of the predictive performances of DeepSEA across different types of experiments. a) Correlation between human (hg19, *x* axis) and mouse (mm39, *y* axis) AUC-PR values (blue-dotted line and statistics). The size of the dots indicates the number of peaks counted in the experiments used to train DeepSEA (Zhou et al. 2018). b and c) Correlations between peak count of experiments used for DeepSEA training and AUC-PR of human b) or mouse c). The colors of the dots represent the type of experiments with TF binding sites (red), DNase (orange), and histone marks (yellow). The correlations take only TF (red-dotted lines and statistics) into account or all experiments (blue-dotted lines and statistics). d) Visualization of the *Sox2* and *Igf2* regions through the IGV. The mouse-annotated genes are shown on the first track with *Sox2* and *Igf2* on the left and right panels, respectively. For CTCF (red), NANOG (blue), DNase (green), and H3K4me3 (purple), both observed experiments in mouse embryonic stem cells (ESC) (top) and predicted annotations (bottom) are shown for comparison. Annotations of the mouse genome are from Ensembl release 112.

Taken together, our results demonstrated 2 levels of analysis depending on TF or chromatin accessibility and histone mark predictions. For TF predictions, our results showed that the amount of data for training (ie the number of peaks) is a critical factor for accurate cross-species predictions, which makes it more difficult to predict TFs that are context-specific. For chromatin accessibility and histone marks, the lower correlations with the amount of data sustain biologically more complex experiments.

### Prediction of chromatin annotations in livestock using human and mouse-trained models

To assess the predictive ability of the neural networks across the phylogenetic tree, we then predicted CTCF, 4 histone marks, and chromatin accessibility experiments in 5 tissues (adipose, liver, lung, muscle, and spleen) with DeepSEA (Zhou et al. 2018) and Enformer (Avsec et al. 2021) from reference genomes of livestock species. For the data analyses, the chosen predictions are mentioned in Supplementary Data 13. We chose 2 mammals (pig, Sscrofa11.1, and cattle, ARS-UCD1.2), 1 bird (chicken, bGalGal1), and 1 fish (European seabass, dlabrax2021). We used the DeepSEA model to compare the metrics between the human, the mouse, and the livestock species genomes. We also used the Enformer model, which is expected to outperform other neural networks given its additional use of transformer layers (Fig. 4; Supplementary Data 14). As for mouse prediction assessment, we considered a bin positive if at least half of the bin overlaps an observed peak; otherwise, it was considered negative.

**Fig. 4.**
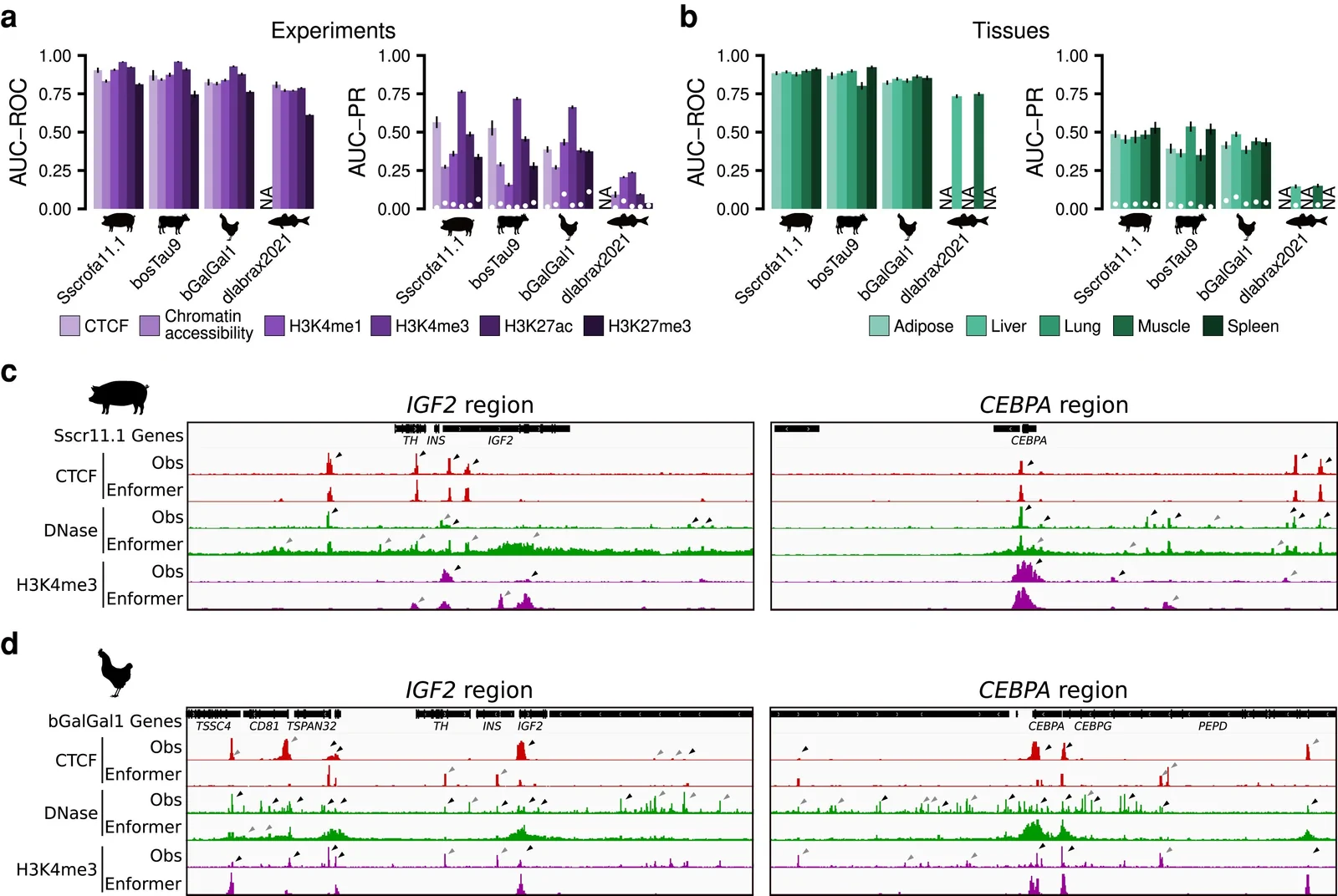
Predictive performances of the Enformer human- and mouse-trained neural network on different livestock genomes. a and b) AUC-ROC (left) and AUC-PR (right) for the same predictions from pigs, cattle, chickens, and European seabass reference genomes with Enformer (Avsec et al. 2021) grouped by experiments (a, purple) or by tissue (b, green). AUC-PR baselines are in white. Data shown (including AUC-PR baselines) are means ± SE. Grouped by experiments, *n* = 36, 30, 44, 52, 34, and 44 for CTCF, chromatin accessibility, H3K4me1, H3K4me3, H3K27ac, and H3K27me3, respectively. Grouped by tissues, *n* = 50, 62, 34, 56, and 38 for the adipose, liver, lung, muscle, and spleen, respectively. c and d) Visualization of the *IGF2* (left) and *CEBPA* (right) regions in pig c) and chicken d) through the IGV. The annotated genes are shown on the first track. For CTCF (red), DNase (green), and H3K4me3 (purple), both observed experiments in pig and chicken liver tissue (top), and predicted experiments (bottom) are shown for comparison. Annotations of pig and chicken genomes are from Ensembl release 112.

First, the AUC-PR metrics obtained using DeepSEA for the comparison between the mouse (Fig. 2a, d, and e) and the 4 species (Supplementary Data 14a) studied varied according to the experiments. The binding of CTCF (0.412 ± 0.031) and DNase (0.287) appeared to be better predicted in mice than in pigs (0.353 ± 0.035 SE and 0.220 ± 0.012 SE), cattle (0.309 ± 0.035 SE and 0.230 ± 0.017 SE), and chickens (0.284 ± 0.010 SE and 0.230 ± 0.021 SE). Similarly, H3K27me3 was better predicted in mice. While mouse (Supplementary Data 10 and 11), pig, and cattle observed data (Supplementary Data 15) show similar peak features, these results can be the consequence of overall low predictive performance. Conversely, H3K4me1, H3K4me3, and H3K27ac in pigs, cattle, and chickens (Supplementary Data 14a) seem to be predicted more accurately than in the mouse (Fig. 2d and e). Second, Enformer showed better predictive ability than DeepSEA, since all AUC-PR values obtained with Enformer (Fig. 4a and b) are higher than those measured with DeepSEA (Supplementary Data 14a and b) for all 4 species and tissues.

We also compared Enformer performances with LiftOver, classically used to transfer genomic annotations between species, to predict CTCF, H3K4me1, and H3K4me3 in the lungs for pig and cattle. As shown in Supplementary Data 16, Enformer can predict 4.4, 26.9, and 38.5 times more bins than LiftOver for CTCF, H3K4me1, and H3K4me3 in pig, and similar results are observed for cattle. Moreover, with a 0.464 mean sensitivity and 0.647 mean precision in pig (CTCF, H3K4me1, and H3K4me3), Enformer also shows better sensitivity and precision than LiftOver (0.053 mean sensitivity and 0.238 mean precision).

Overall, we observed more variability in the prediction metrics across different experiments than between different tissues (Fig. 4a and b; Supplementary Data 14a and b and 17e and h), as well as better performances than a random classifier (Supplementary Data 17a to d). However, AUC-PR values are lower in cattle adipose, liver, and muscle tissues than in pig and chicken tissues. While data quality and processing are not excludable, our data show similar features between species (Supplementary Data 15), sustaining metabolic specificities of those tissues in ruminants (Fig. 4b). Indeed, monogastric and ruminant energetic metabolism evolved differently due to their respective energetic substrate (glucose and short fatty acids), as reviewed in (Bauchart et al. 1996; Hocquette and Bauchart 1999). Thus, our results seem to support this by showing particular chromatin regulation related to energetic metabolism in cattle (Chen et al. 2022), which might be the consequence of no ruminant data in the training dataset. For nonmammalian genomes, although we found slightly less good metrics for the chicken genome, much lower metrics for the European seabass genome were found, suggesting a shift in the predictive ability of neural networks across phylogeny (Fig. 4a and b).

To show whether Enformer (Avsec et al. 2021) is able to predict species-specific peaks or is limited to orthologous peaks, we compared the distribution of prediction scores between bins overlapping peaks lifted over the human genome (ie orthologous peaks). As shown in Supplementary Data 18, prediction scores are significantly different between orthologous and nonorthologous bins. However, the distributions of nonorthologous bins show 2 clear groups: one group with high prediction scores, similar to the prediction scores of orthologous bins, and one group with low prediction scores corresponding to divergent species-specific peaks. This shows that among nonorthologous bins, many failed to be predicted as positive with LiftOver but could be predicted as positive with deep learning. We also observed that the distributions are similar across all species, while less than 5% of chicken peaks have been lifted over the human genome, which is lower than 35% to 42% lifted mammalian peaks, sustaining interest in the cross-species approach for nonmammalian species.

To illustrate predictions, we focused on 2 genomic regions: the *IGF2* locus, which undergoes genomic imprinting in pigs (Fig. 4c) but not in chickens (Fig. 4d) (Frésard et al. 2014), and the *CEBPA* region, which is a master TF gene for adipogenesis (Lefterova et al. 2008). Overall, as previously observed from genome-wide AUC-PR values, the Enformer neural network showed better predictive ability than the DeepSEA model (Fig. 4c and d; Supplementary Data 14c and d). For CTCF predictions in the IGF2 region, all peaks have been correctly predicted in pigs and half of the peaks in chickens (Fig. 4c and d). Conversely, more false positives are observed for H3K4me3 peaks in pigs than in chickens in both regions, while the opposite is shown for DNase (Fig. 4c and d).

Our results revealed that cross-species chromatin predictions based on neural networks trained on human data are better than classical tools, such as LiftOver (Supplementary Data 16 and 18), and go beyond mammals, especially for CTCF, H3K4me3, and H3K27ac (Fig. 4; Supplementary Data 14 and 18).

### Prediction performances regarding the phylogeny and sequence conservation

In order to better understand the relationship between predictive annotation metrics across species, we first evaluated the correlation between the AUC-PR of H3K4me3 and the phylogenetic distance calculated as substitutions per site (Fig. 5a to c). The differences of DeepSEA AUC-PR values measured among the species seem explained by the phylogenetic distances (Fig. 5a), since strong correlations are obtained between the 4 livestock species, mouse, and human (Fig. 5b). A similar trend is observed for the Enformer neural network (Fig. 5c). This suggests stable prediction performances from mammals to chickens. However, beyond 1.166 substitutions per site, with only the seabass showing lower AUC-PR, we are not able to conclude about the prediction performances across phylogeny.

**Fig. 5.**
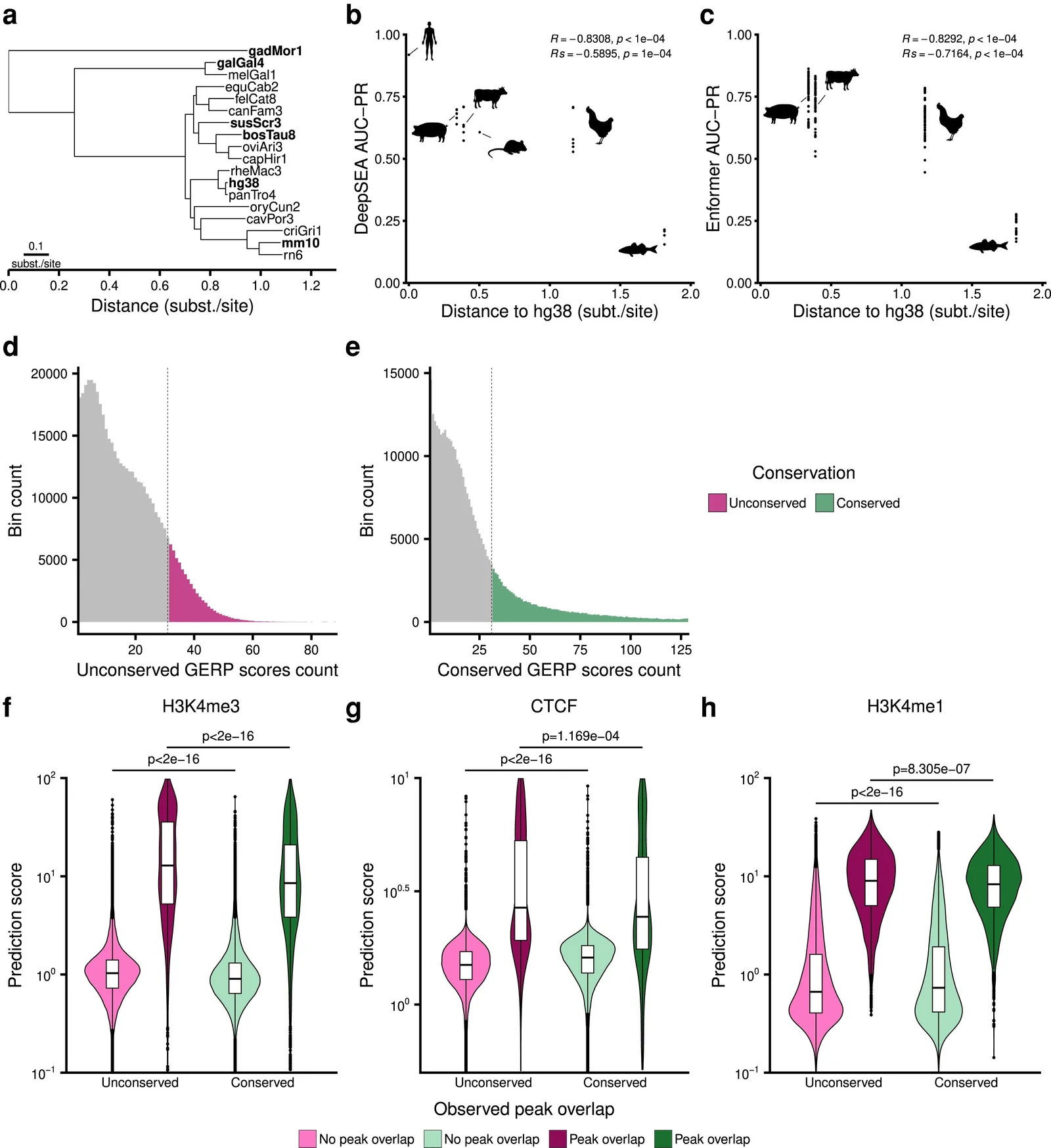
Impact of the sequence conservation on the performance of predictions in pigs. a) Phylogenetic distance (substitutions per site) of commonly studied species and species of interest (bold). gadMor1 is used as a proxy for European seabass. b and c) Relationship between AUC-PR of H3K4me3 predicted by DeepSEA (Zhou et al. 2018) b) and Enformer (Avsec et al. 2021) c) and phylogenetic distances, relative to the human genome, of species of interest. Pearson (*R*) and Spearman (*R*s) correlations have been calculated, excluding human AUC-PR. d and e) Distribution of the number of unconserved d) and conserved e) nucleotides per bin. Ten percent of nonoverlapping exon bins has been sampled in each chromosome of the pig reference genome (*n* = 1,784,013). Each nucleotide has been classified as unconserved or conserved, based on its GERP score relative to the whole-genome distribution of GERP scores. For each bin, the number of unconserved and conserved nucleotides has been extracted. For d and e, the vertical line is the quarter of bin size used to classify unconserved (pink, *n* = 52,403) or conserved (green, *n* = 68,938) bins. f to h) Distribution of Enformer prediction scores of H3K4me3 f), CTCF g), and H3K4me1 h) in the adipose tissue according to their conservation status (pink vs green) and the overlap (dark pink or green) or not (light pink or green) with an observed peak. For each experiment, a 2-sided unpaired Wilcoxon test has been realized between unconserved not overlapping a peak bins (light pink, *n* = 50,907, 51,704, and 49,956 for H3K4me3, CTCF, and H3K4me1, respectively) and conserved not overlapping a peak bins (light green, *n* = 60,180, 67,984, and 65,002 for H3K4me3, CTCF, and H3K4me1, respectively), and between unconserved overlapping a peak bins (dark pink, *n* = 1,496, 699, and 2,447 for H3K4me3, CTCF, and H3K4me1, respectively) and conserved overlapping a peak bins (dark green, *n* = 2,758, 954, and 3,936 for H3K4me3, CTCF, and H3K4me1, respectively). Additionally, r effect sizes have been calculated for each group: *r* = 0.082, 0.143, 0.149, 0.095, 0,035, and 0.062 for H3K4me3 no peak overlap, H3K4me3 peak overlap, CTCF no peak overlap, CTCF peak overlap, H3K4me1 no peak overlap, and H3K4me1 peak overlap groups, respectively.

In addition, we used a metric for sequence conservation—the Genomic Evolutionary Rate Profiling (GERP) scores calculated from 91 mammal genomes—to investigate the impact of conserved and unconserved sequences on prediction performances (Fig. 5d to h). First, we computed the GERP score of the 10th and 90th percentiles of whole-genome GERP scores, and then we down-sampled 10% of bins, not overlapping exons, from each chromosome of the pig genome. Nucleotides with a GERP score lower than or equal to the 10th percentile have been classified as unconserved, and nucleotides with a GERP score greater than or equal to the 90th percentile have been classified as conserved. Then, for each sampled bin, we counted the number of unconserved and conserved nucleotides. Finally, we evaluated the distribution of unconserved (Fig. 5d) and conserved (Fig. 5e) nucleotides in the bins. We showed that a high proportion of bins have a low proportion of unconserved (Fig. 5d), as well as conserved (Fig. 5e), nucleotides. We classified bins as unconserved or conserved when at least a quarter of their nucleotides (≥32 nucleotides) were unconserved or conserved, respectively. We then split the bins into 2 classes, depending on whether or not they overlapped with an observed peak (Fig. 5f to h). For H3K4me3 (Fig. 5f), we show that a statistical difference exists between conserved and unconserved sequences, whether bins overlap an observed peak or not (*P* < 2.10^−16^ on a 2-sided unpaired Wilcoxon test). However, the differences measured between classes were negligible (0.143 and 0.082 for bins overlapping a peak or not, respectively). The same comparisons for CTCF (Fig. 5g) and H3K4me1 (Fig. 5h) showed the same tendency as H3K4me3 (Fig. 5f).

Overall, our results showed that cross-species neural network prediction accuracy goes beyond sequence conservation (Fig. 5; Supplementary Data 16 and 18).

### Prediction performances in pigs with respect to sequence ontology

We then assessed whether prediction performances depend on sequence ontology. For this purpose, we predicted 6 experiments in 5 tissues on the pig reference genome and evaluated AUC-ROC and AUC-PR metrics depending on different genomic features (Fig. 6a).

**Fig. 6.**
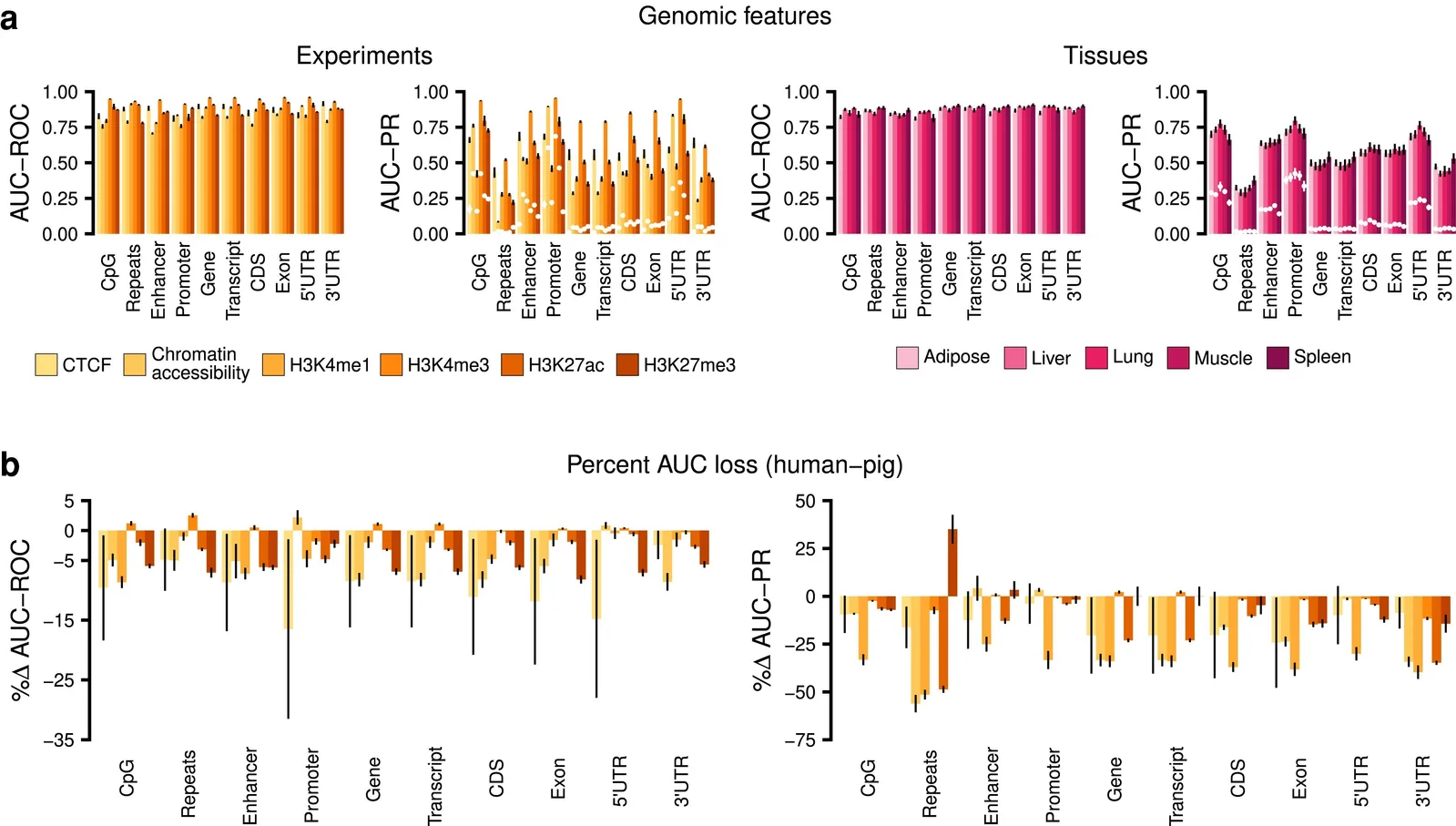
Impact of pig-annotated genomic features on the performance of Enformer predictions. a) AUC-ROC (left) and AUC-PR (right) calculated for 8 genomic features between observations from the FAANG project and predictions from the pig reference genome with Enformer (Avsec et al. 2021), grouped by experiments (yellow) or tissues (pink). AUC-PR baselines are in white. Data shown (including AUC-PR baselines) are means ± SE. Grouped by experiments, for each genomic feature, *n* = 36, 30, 44, 52, 34, and 44 for CTCF, chromatin accessibility, H3K4me1, H3K4me3, H3K27ac, and H3K27me3, respectively. Grouped by tissues, for each genomic feature, *n* = 50, 62, 34, 56, and 38 for the adipose, liver, lung, muscle, and spleen, respectively. b) Difference in percentage of AUC-ROC (left) and AUC-PR (right) from human (GRCh38/hg38) to pig (Sscrofa11.1) muscle. For each genomic feature, 1 human AUC has been subtracted from pig AUCs (*n* = 2, 6, 12, 14, 10, and 12 for CTCF, chromatin accessibility, H3K4me1, H3K4me3, H3K27ac, and H3K27me3, respectively). Data shown are means ± SE.

Here again, we observed that all AUC-PR showed higher values than their own reference baseline (ie prevalence) and random classifier, regardless of the genomic feature (Fig. 6a, Supplementary Data 19). We found that promoters, CGIs, and 5′UTR regions are particularly well predicted with AUC-PR values above 0.564 for all experiments, except H3K4me1, which has a mean AUC-PR of 0.459 ± 0.158 SE, 0.422 ± 0.172 SE, and 0.474 ± 0.141 SE for promoters, CpG, and 5′UTR, respectively (Fig. 6a). In contrast, repeated sequences and 3′UTR regions showed AUC-PR under 0.521 for all experiments, except CTCF and H3K4me3 in 3′UTR, which, respectively, capped at 0.640 ± 0.221 SE and 0.616 ± 0.091 SE (Fig. 6a). With promoters, enhancers are the main chromatin regulatory features, but they display lower sequence conservation compared to promoters (Villar et al. 2015). Our results show that they are slightly less well predicted than promoters, CpG, and 5′UTR but better predicted than the similarly predicted CDS and exons. Indeed, the results showed similar AUC-PR for H3K4me3, H3K27ac, and H3K27me3 marks for enhancers, exons, and CDS, but they showed better AUC-PR for CTCF (0.685 ± 0.192 SE, 0.562 ± 0.190 SE, and 0.543 ± 0.174 SE for enhancers, exons, and CDS, respectively), chromatin accessibility (0.526 ± 0.073 SE, 0.479 ± 0.076 SE, and 0.425 ± 0.071 SE), and H3K4me1 (0.510 ± 0.141 SE, 0.401 ± 0.136 SE, and 0.425 ± 0.143 SE) (Fig. 6a).

In addition, we measured the difference in prediction performance between human and pig genomes in muscle tissues (Fig. 6b). The results showed that the most conserved regulatory features (promoters, CpG, 5′UTR) seem to have the least AUC-PR loss (Fig. 6b). Moreover, the regulation pattern of promoter activity seemed well predicted by the neural networks, as shown by poor AUC-PR loss of H3K4me3 and DNase-seq. In contrast, the enhancers seem well predicted, but their pattern seems not fully understood (Fig. 6a). Indeed, the enhancer annotations from Ensembl are based on H3K4me1 and H3K27ac colocalization, but with AUC-PR rising to 0.510 ± 0.141 and 0.640 ± 0.090, respectively, our results show moderate predictions on those marks in the enhancers. While H3K27ac can be found not only in enhancers but also in promoters, H3K4me1 is mostly known as an enhancer-repressive mark. However, our results show that H3K4me1 is best predicted in enhancers over all features (Fig. 6a) and, with a delta AUC-PR of −25.0%, they also show the least loss from human to pig (Fig. 6b), suggesting a better association of this mark with enhancers. Those results are in concordance with DeepGCF (Li et al. 2023) functional conservation scores and suggest that neural networks improve the overall predictions. However, for H3K27me3, pig performances overlapping repeat features seem better predicted in pig than in human. This is likely due to high variability and the poor annotations of repeat features in the pig genome.

Altogether, our results showed that prediction performances vary across genomic features, with promoters, CGs, and 5′UTR regions being the most accurately predicted, but results are encouraging for the analysis of less conserved sequences such as enhancers.

## Discussion

Here, we demonstrated the accuracy of neural networks trained with human and mouse data to predict annotations on genomes of livestock species. Extensive benchmarking revealed a drop in prediction performances from the human genome used for training neural networks to genomes from the same phylogenetic clade (mammals) but, strikingly, similar performances for a more distant clade (Aves). The results suggested a breakpoint at a phylogenetic distance beyond 1.166 substitutions per site, as the prediction metrics for the seabass genome were very low. However, this result is based on a single species, and the analysis of additional species is needed to support such a conclusion. Such results are expected as models are not perfect. However, by predicting better than homology-based methods (ie LiftOver), we also showed that they are not limited to orthologous regions, underlying conserved predictive motifs over sequence homology. Nonetheless, this demonstrates the potential of such methods and how we can take advantage of the availability of data from well-studied species to improve our understanding of less-studied species.

A few studies have highlighted conservation of regulatory elements in mammals (Woo and Li 2012; Villar et al. 2015), vertebrates (Pérez-Rico et al. 2017; Phan et al. 2025), and other animals (Wong et al. 2020). For example, Wong et al. showed that while sequences may be partially conserved between human, mouse, zebrafish, and sponge, clusters of TF binding motifs are conserved from the human up to the zebrafish (Wong et al. 2020). These fundamental results demonstrate that the function of gene regulatory elements, such as enhancers, is maintained over the course of evolution regardless of their sequence conservation (Yang et al. 2015; Snetkova et al. 2021). In parallel, during the last 2 decades, computational methods based on sequence conservation have been used for cross-species prediction, but are inherently not appropriate for unconserved sequences (Davydov et al. 2010; Pollard et al. 2010; Zhao et al. 2014). For example, GERP++ relies on the alignment of the genome sequences of many divergent species and searches for sequences whose similarity has been maintained during evolution (Davydov et al. 2010). In contrast, more recently, machine learning methods, which do not rely on sequence conservation but instead on sequence features such as DNA motifs, have shown that enhancers share functional properties across species, supporting biological data, but this was often limited to a few species of mammals (Chen et al. 2018; MacPhillamy et al. 2022; Kaplow et al. 2023; Li et al. 2023). For instance, the 2-step neural network approach, DeepGCF (Li et al. 2023), first predicts epigenomic functional effects and then uses these effects as input to predict the functional conservation between humans and pigs. Here, our findings are in concordance with the literature, supporting that we can predict chromatin regulation beyond mammals, regardless of sequence conservation.

Although we were able to evaluate the ability of neural networks to predict across several species and point out strengths as well as some limits, our analysis was limited by the data available in FAANG for the evaluated livestock species. It would be valuable to study intermediate species across the tree of life, such as reptiles and amphibians, to accurately define the shift in chromatin prediction annotation abilities. However, accessing valuable chromatin-omics data for experimental purposes may present an obstacle to performing AUC-PR. In addition, it would be of great interest to evaluate such performances for an even broader range of experiments. For example, the evaluation of other histone modifications would have been interesting, such as H3K9me3, which is enriched for silencing LTRs, LINEs, and SVAs repeated sequences, and with tissue specificities (Trizzino et al. 2018).

As pointed out here and by the literature, machine and deep learning models are not perfect, and several options exist in order to improve the cross-species predictions. Over Enformer and its use of Transformer layers, it has been shown that model architecture is a key element in highlighting different features and interactions between them (Chen et al. 2018; Cochran et al. 2022). Moreover, Kelley showed that using multispecies data during the deep learning model training phase improves predictions (Kelley 2020), and while annotation data from not commonly studied species are often lacking, alternatives like pseudolabeling have been proposed (Phan et al. 2024). Finally, the improvement of molecular techniques used to study chromatin regulation and annotate genomes might have some biases (sequence affinity of enzymes, for example). Novel innovative methods, such as CUT&Tag, for efficient epigenomic profiling, that have not been used in the training set nor in the evaluation set, may likely improve predictions of chromatin accessibility (Kaya-Okur et al. 2019). Here, we used 3 artificial neural networks relying on different parameters (DeepBind (Alipanahi et al. 2015), DeepSEA (Zhou et al. 2018), and Enformer (Avsec et al. 2021)) as a first step to evaluate their abilities for cross-species predictions of chromatin annotation, mainly in livestock species. This area of research is in constant evolution, and novel models are regularly released. As an example, ChromBPNet (Pampari et al. 2025) seems to show prediction performances close to Enformer performances but with a lighter architecture based on convolution layers. While it has not been used for cross-species predictions, this suggests that other models might be used in order to improve cross-species predictions. However, this model (Pampari et al. 2025) is dedicated to the prediction of TFBS, so we chose not to include it in our analyses.

In summary, our research opens the field of predicting experiments for species of agronomical interest with artificial neural networks trained with human and mouse data. While neural networks are generally developed to predict a variant impact in human pathologies, we show here that we can also use them for cross-species predictions and consider their use in the study of complex traits for species of agronomical interest.

## Code and data availability

All the available data from ENCODE, ReMap2022, and FAANG used in this article are mentioned in Supplementary Data 2 to 4. All the code is available at https://forge.inrae.fr/genesis/EAGLE. All the bigwig prediction files are available at https://entrepot.recherche.data.gouv.fr/ under the accession numbers https://doi.org/10.57745/TN3R6F, https://doi.org/10.57745/92QIQS, and https://doi.org/10.57745/EEQDSP.

Supplemental material available at G3 online.

## Supplementary Material

jkag230_Supplementary_Data
